# Association of Prolactin, Oxytocin, and Homocysteine With the Clinical and Cognitive Features of a First Episode of Psychosis Over a 1-Year Follow-Up

**DOI:** 10.1093/ijnp/pyad051

**Published:** 2023-08-21

**Authors:** Maria Hidalgo-Figueroa, Alejandro Salazar, Cristina Romero-López-Alberca, Karina S MacDowell, Borja García-Bueno, Miquel Bioque, Miquel Bernardo, Mara Parellada, Ana González-Pinto, M Paz García-Portilla, Antonio Lobo, Roberto Rodriguez-Jimenez, Esther Berrocoso, Juan C Leza, FLAMM-PEPs study, CIBERSAM

**Affiliations:** Centro de Investigación Biomédica en Red de Salud Mental (CIBERSAM), ISCIII, Madrid, Spain; Biomedical Research and Innovation Institute of Cadiz (INiBICA), Research Unit, Puerta del Mar University Hospital, Cádiz, Spain; Neuropsychopharmacology and Psychobiology Research Group, Psychobiology Area, Department of Psychology, Universidad de Cádiz, Puerto Real (Cádiz), Spain; Biomedical Research and Innovation Institute of Cadiz (INiBICA), Research Unit, Puerta del Mar University Hospital, Cádiz, Spain; Department of Statistics and Operational Research, University of Cádiz, Puerto Real (Cádiz), Spain; The Observatory of Pain, University of Cádiz, Cádiz, Spain; Centro de Investigación Biomédica en Red de Salud Mental (CIBERSAM), ISCIII, Madrid, Spain; Biomedical Research and Innovation Institute of Cadiz (INiBICA), Research Unit, Puerta del Mar University Hospital, Cádiz, Spain; Personality, Evaluation and Psychological Treatment Area, Department of Psychology, Universidad de Cádiz, Puerto Real (Cádiz), Spain; Centro de Investigación Biomédica en Red de Salud Mental (CIBERSAM), ISCIII, Madrid, Spain; Departamento de Farmacología y Toxicología, Facultad de Medicina, Univ. Complutense de Madrid, Instituto de Investigación Sanitaria Hospital 12 de Octubre (i+12), IUINQ, Madrid, Spain; Centro de Investigación Biomédica en Red de Salud Mental (CIBERSAM), ISCIII, Madrid, Spain; Departamento de Farmacología y Toxicología, Facultad de Medicina, Univ. Complutense de Madrid, Instituto de Investigación Sanitaria Hospital 12 de Octubre (i+12), IUINQ, Madrid, Spain; Centro de Investigación Biomédica en Red de Salud Mental (CIBERSAM), ISCIII, Madrid, Spain; Institut d’investigacions Biomèdiques August Pi i Sunyer (IDIBAPs), Barcelona Clínic Schizophrenia Unit (BCSU), Neuroscience Institute, Hospital Clínic de Barcelona, Department of Medicine, University of Barcelona, Barcelona, Spain; Centro de Investigación Biomédica en Red de Salud Mental (CIBERSAM), ISCIII, Madrid, Spain; Institut d’investigacions Biomèdiques August Pi i Sunyer (IDIBAPs), Barcelona Clínic Schizophrenia Unit (BCSU), Neuroscience Institute, Hospital Clínic de Barcelona, Department of Medicine, University of Barcelona, Barcelona, Spain; Centro de Investigación Biomédica en Red de Salud Mental (CIBERSAM), ISCIII, Madrid, Spain; Department of Child and Adolescent Psychiatry, Institute of Psychiatry and Mental Health, Hospital General Universitario Gregorio Marañón, IiSGM, School of Medicine, Universidad Complutense, Madrid, Spain; Centro de Investigación Biomédica en Red de Salud Mental (CIBERSAM), ISCIII, Madrid, Spain; Department of Psychiatry, Hospital Universitario de Alava, BIOARABA, EHU, Vitoria-Gasteiz, Spain; Department of Psychiatry, Universidad de Oviedo, Instituto de Investigación Sanitaria del Principado de Asturias (ISPA), Instituto de Neurociencias del Principado de Asturias (INEUROPA), Servicio de Salud del Principado de Asturias (SESPA), Oviedo, Spain; Centro de Investigación Biomédica en Red de Salud Mental (CIBERSAM), ISCIII, Madrid, Spain; Department of Medicine and Psychiatry, Universidad de Zaragoza, Instituto de Investigación Sanitaria Aragón (IIS Aragón), Zaragoza, Spain; Centro de Investigación Biomédica en Red de Salud Mental (CIBERSAM), ISCIII, Madrid, Spain; Department of Psychiatry, Instituto de Investigación Sanitaria Hospital 12 de Octubre (imas12)/Universidad Complutense de Madrid (UCM), Madrid, Spain; Centro de Investigación Biomédica en Red de Salud Mental (CIBERSAM), ISCIII, Madrid, Spain; Biomedical Research and Innovation Institute of Cadiz (INiBICA), Research Unit, Puerta del Mar University Hospital, Cádiz, Spain; Neuropsychopharmacology and Psychobiology Research Group, Psychobiology Area, Department of Psychology, Universidad de Cádiz, Puerto Real (Cádiz), Spain; Centro de Investigación Biomédica en Red de Salud Mental (CIBERSAM), ISCIII, Madrid, Spain; Departamento de Farmacología y Toxicología, Facultad de Medicina, Univ. Complutense de Madrid, Instituto de Investigación Sanitaria Hospital 12 de Octubre (i+12), IUINQ, Madrid, Spain

**Keywords:** First-episode psychosis, prolactin, oxytocin, homocysteine, cognition

## Abstract

**Background:**

The clinical debut of schizophrenia is frequently a first episode of psychosis (FEP). As such, there is considerable interest in identifying associations between biological markers and clinical or cognitive characteristics that help predict the progression and outcome of FEP patients. Previous studies showed that high prolactin, low oxytocin, and high homocysteine are factors associated with FEP 6 months after diagnosis, at which point plasma levels were correlated with some clinical and cognitive characteristics.

**Methods:**

We reexamined 75 patients at 12 months after diagnosis to measure the evolution of these molecules and assess their association with clinical features.

**Results:**

At follow-up, FEP patients had lower prolactin levels than at baseline, and patients treated with risperidone or paliperidone had higher prolactin levels than patients who received other antipsychotic agents. By contrast, no changes in oxytocin and homocysteine plasma levels were observed between the baseline and follow-up. In terms of clinical features, we found that plasma prolactin and homocysteine levels were correlated with the severity of the psychotic symptoms in male FEP patients, suggesting that they might be factors associated with psychotic symptomatology but only in men. Together with oxytocin, these molecules may also be related to sustained attention, verbal ability, and working memory cognitive domains in FEP patients.

**Conclusion:**

This study suggests that focusing on prolactin, oxytocin, and homocysteine at a FEP may help select adequate pharmacological treatments and develop new tools to improve the outcome of these patients, where sex should also be borne in mind.

**Significance Statement**

This work analyses the association between biological markers and clinical/cognitive characteristics in first-episode psychosis (FEP), which is often the initial debut of schizophrenia. Previous studies identified high prolactin, low oxytocin, and high homocysteine levels as factors associated with a FEP at baseline. The current study reexamined these patients 6 months later to measure changes in these molecules and assess their relationship with clinical features. The results showed that FEP patients had lower prolactin levels at follow-up, but those treated with risperidone or paliperidone had higher levels than those on other antipsychotics. No significant changes were observed in oxytocin or homocysteine levels. Interestingly, plasma prolactin and homocysteine levels were correlated with the severity of psychotic symptoms in male patients, suggesting a sex-specific relationship. The study highlights the potential importance of monitoring these molecules in FEP patients to select appropriate pharmacological treatments and improve patient outcomes, where sex should also be considered.

## INTRODUCTION

Schizophrenia is a serious illness with a multifactorial etiology that includes interactions between genetic vulnerability and environmental factors (Stilo and Murray, 2019). The onset of this disorder is usually in late adolescence or early adulthood, when its clinical debut is characterized by psychosis. Around 3% of the population suffers a first episode of psychosis (FEP), whereas the prevalence of schizophrenia in the general population is closer to 1% (Lichtermann et al., 2000; Perälä et al., 2007), indicating that schizophrenia is only one possible diagnosis after an FEP. Indeed, the outcome of these patients is variable and ranges from total remission to chronicity (Peralta et al., 2021). It is estimated that after an FEP, up to 80% of the patients will suffer a psychotic relapse in the next 5 years (Alvarez-Jimenez et al., 2012) such that understanding the factors that induce relapse would be valuable for the diagnosis, prognosis, and treatment of patients. Early intervention is essential to improve the prognosis of the disorder (Alvarez-Jimenez et al., 2012; Galletly et al., 2016; Peralta et al., 2021). Thus, it is clearly important to study the associations of biological molecules with clinical and cognitive features of an FEP, which may help to predict the outcome of the patients and identify more adequate treatments.

We previously found alterations to plasma levels of the hormones prolactin and oxytocin at early stages of FEP, resulting in higher prolactin and lower oxytocin levels compared with healthy controls (Hidalgo-Figueroa et al., 2022). Prolactin plays a notable role in lactation and is involved in several activities controlled by the brain, such as energy balance, anxiety, stress regulation, food intake, and maternal behavior (Patil et al., 2014; Delgado-Alvarado et al., 2019). Antipsychotic medication usually elevates this hormone in patients with schizophrenia, yet unmedicated patients also have higher prolactin levels than healthy control individuals (Garcia-Rizo et al., 2012; Riecher-Rössler et al., 2013; Petrikis et al., 2016; Delgado-Alvarado et al., 2019; Pisk et al., 2019; Studerus et al., 2021). The elevation of prolactin may be dampened by the use of second-generation antipsychotic agents such as ziprasidone, clozapine, olanzapine, aripiprazole, or quetiapine (Kane et al., 1981; Beasley et al., 1996; Daniel and Copeland, 2000; Crespo-Facorro et al., 2017; Wadoo et al., 2017), although other second-generation antipsychotics maintain the increase in prolactin levels, such as paliperidone, risperidone, or amisulpride (Berwaerts et al., 2010). Some consequences of long-term exposure to antipsychotic drugs that increase prolactin levels were recently described, such as a greater risk of breast cancer and low-energy fractures, indicating that hyperprolactinemia needs to be monitored in these patients (Taipale et al., 2021; Solmi et al., 2022). We previously showed that patients on any antipsychotic treatment had higher plasma prolactin levels during an FEP than unmedicated patients and control participants, and those with more severe symptoms were prescribed (Hidalgo-Figueroa et al., 2022). Thus, efforts must be made to understand whether increased prolactin during an FEP occurs specifically in more severe cases or if it is just a side effect of antipsychotic drug administration. Indeed, high prolactin has been correlated with the severity of an FEP in male but not female patients, suggesting that sex-dependent phenomena can potentially offer some protection in females (González-Blanco et al., 2016; Delgado-Alvarado et al., 2019; Labad, 2019; Hidalgo-Figueroa et al., 2022). However, the clinical significance of this difference remains controversial, specifically when considering the association between prolactin and cognitive performance, as increased levels of this hormone were associated with impaired cognition at the early stages of psychosis in a group of men and women (Montalvo et al., 2014). Nevertheless, we found better cognitive performance in male patients with higher prolactin levels, specifically in an attention task (Hidalgo-Figueroa et al., 2022), indicating that further sex-disaggregated analyses should be performed to fully elucidate the possible relationship between prolactin levels and cognition.

Oxytocin influences social behavior, emotion, and learning, and changes in its levels have been specifically related to neuropsychiatric conditions (Cochran et al., 2013; Kania et al., 2020). Altered plasma oxytocin levels have also been described in patients with pathologies in which social behaviors deteriorate, such as autism or schizophrenia (Cochran et al., 2013; Kirsch, 2015; Carrasco et al., 2020). In both FEP and schizophrenia patients, lower oxytocin levels were detected, although the clinical significance of this remains unclear. By contrast, low oxytocin levels in patients with schizophrenia were related to the severity of negative symptoms (Shilling and Feifel, 2016), a relationship not found in FEP patients (Rubin et al., 2013; Hidalgo-Figueroa et al., 2022). Although there is insufficient evidence that the oxytocinergic system is altered in patients with psychosis and of its relationship with clinical and cognitive symptoms (Rutigliano et al., 2016), oxytocin levels in FEP patients do appear to be associated with cognitive performance in a sex-dependent manner (Hidalgo-Figueroa et al., 2022). Indeed, whereas low oxytocin in women was associated with poor sustained attention, in men it was related to better performance in this cognitive domain. To understand the relationship between oxytocin and clinical/cognitive phenomenon, longitudinal studies and sex-disaggregated analysis are clearly necessary (Williams and Bürkner, 2017; Zheng et al., 2019; Hidalgo-Figueroa et al., 2022).

In addition to prolactin and oxytocin, we previously identified homocysteine as a potential risk factor at the beginning of an FEP (García-Bueno et al., 2014). Homocysteine is a sulphur-containing amino acid formed through metabolism of the essential amino acid methionine. Hyperhomocysteinemia has been related to vascular and neuronal damage, and it influences neuronal functions though different signaling pathways (Kruman et al., 2000; Ho et al., 2002; Luzzi et al., 2022). In addition, higher plasma homocysteine levels may increase the risk of schizophrenia by 70% (Muntjewerff et al., 2006), and levels have been associated with the severity of negative symptoms (Petronijević et al., 2008; Fan et al., 2017). However, to our knowledge, the evolution of homocysteine levels in later stages after an FEP, or its correlation to clinical or cognitive symptoms, has yet to be described.

In short, prolactin, oxytocin, and homocysteine plasma levels are altered in FEP patients when assessed soon after diagnosis. To determine whether these molecules could serve as potential biomarkers and therapeutic targets at the onset of psychosis, longitudinal studies in sex-disaggregated patients and further correlations with specific clinical and cognitive variables will be necessary. Here we analyzed the levels of plasma prolactin, oxytocin, and homocysteine in patients during an FEP both at baseline (up to 6 months after inclusion) and after a 1-year follow-up. We also studied their associations with clinical characteristics and cognitive performance.

## METHODS

### Participants

The FEP patients included in this study were from Flamm-PEPs, a multicenter study performed by the Spanish Network for Mental Health Research (CIBERSAM). The patient inclusion/exclusion criteria and the clinical protocol were previously published (Bernardo et al., 2013; Garcia-Bueno et al., 2014, 2015). Briefly, the inclusion criteria were (1) aged between 9 and 35 years; (2) beginning and duration of the psychotic symptoms for less than 1 year; and (3) correctly speak Spanish. The exclusion criteria were (1) intellectual disability; (2) IQ <70, together with impaired functioning; (3) history of traumatic head injury with a loss of consciousness; and (4) history of organic disease with mental repercussions. A total of 120 FEP patients were included at baseline (up to 6 months after inclusion), and 75 of them were followed for up to 12 months after inclusion. The ethics committees of all the participating centers approved this study, and all the participants provided their written, informed consent (Garcia-Bueno et al., 2014, 2015).

### Clinical Assessment

DSM-IV-TR (American Psychiatric Association, 2000) criteria were used to diagnose FEP, and the Kiddie-Schedule for Affective Disorders and Schizophrenia Present and Lifetime Version was used for patients younger than 18 years (Kaufman et al., 1997). The severity of the psychotic symptoms was evaluated with the Positive and Negative Syndrome Scale (PANSS) (Kay et al., 1987; Peralta and Cuesta, 1994), whereas the severity of symptoms and the level of functioning were assessed with the Global Assessment of Functioning Scale (GAF) (Endicott et al., 1976) and the Children’s Global Assessment Scale (Shaffer et al., 1983). The potency equivalents relative to chlorpromazine of the daily antipsychotic drug dosage were calculated in accordance with the international consensus (Gardner et al., 2010).

### Cognitive Assessment

Patients completed a cognitive battery following the National Institute of Mental Health MATRICS consensus (Green et al., 2004; Carter et al., 2008). For the purposes of the current study, the following tests were considered: premorbid-IQ through the verbal ability scores of the Vocabulary subtest of WISC-IV for children (Wechsler, 2003) and WAIS-III for adults (Wechsler, 1955); Digit and Letters and Numbers subtest to measure working memory, WAIS-III for adults and WISC-IV for children; processing speed with the Trail Making Test Form A; Trail Making Test Form B (Reitan and Wolfson, 1993) to measure executive functions; and sustained attention through the Continuous Performance Test-II (Conners and Staff, 2004), corrected for age and educational level. Higher scores in verbal ability and working memory tasks are related to better performance in these domains, whereas higher scores in the processing speed, executive functions, and attention tasks are indicative of worse performance in these cognitive domains.

### Plasma Samples Isolation

After overnight fasting, 10 mL of venous blood samples was collected between 8:00 and 10:00 am. The blood was centrifuged at 641*g* for 10 minutes at 4ºC, and the resulting plasma was stored at −80ºC until use.

### Biochemical Measurements in Plasma

#### Prolactin

This hormone was measured using an ELISA kit (ref. 500730, Cayman Chemical, Tallinn, Estonia) following the manufacturer’s instructions and measuring absorbance at 450 nm (Synergy 2, Biotek, Winooski, Vermont, US). The intra- and inter-assay coefficients of variation (CVs) were 3.7% and 4.6%, respectively. Hyperprolactinemia is considered when prolactin exceeds 20 ng/mL in men and 25 ng/mL in women (Halbreich et al., 2003).

#### Oxytocin

Oxytocin was quantified using a commercial competitive ELISA kit (ref. 500440, Cayman Chemical) following the manufacturer’s instructions. To remove molecules that could interfere with the assay (Szeto et al., 2011) and obtain valid results, the plasma samples were previously purified on C18 Sep-Pak columns (Waters, UK). Absorbance was measured at 405 nm (Synergy 2, Biotek), and the intra- and inter-assay CVs were 7.2% and 7.0%, respectively.

#### Homocysteine

This amino acid was quantified using an ELISA kit (ref. CED984Ge, Cloud-Clone, Katy, Texas, US) following the manufacturer’s instructions. Absorbance was measured at 450 nm (Synergy 2, Biotek), and the intra- and inter-assay CVs were <10% and <12%, respectively.

### Statistical Analysis

The characteristics of the sample were described through the absolute and relative (%) frequencies, or mean and SD, depending on the nature of the variable. For each variable, a value was considered to be an outlier if its distance to the box-plot was higher than 1.5 times the interquartile range. Applying this criterion, the outliers were identified and removed from the analyses. Normality was checked with the Kolmogorov-Smirnov test. The differences in the demographic and clinical characteristics of the patients at baseline and at follow-up were analyzed using an unpaired Student *t* test or Mann-Whitney U test in the case of quantitative variables (depending on their nature). For qualitative variables, chi-squared tests were used, assessing the likelihood ratio when more than 20% of the expected values were ≥5. The differences in terms of the biological markers at baseline and follow-up in FEP patients are presented in a plot, showing the mean differences and SEM. Mann-Whitney U tests or *t* tests were used to compare between the groups. The differences in prolactin levels depending on the drug used (none, risperidone/paliperidone, others) were analyzed by ANOVA, applying Bonferroni post hoc comparisons.

The clinical and neuropsychological evaluation of FEP patients is presented as the means ± SD and assessed specifically for men and women. The comparison between the baseline and follow-up in FEP patients was assessed with a *t* test or Mann Whitney U test. Pearson (normal) or Spearman rank (nonnormal) correlation coefficients were used to check the relationships between biological markers and clinical/cognitive variables. Three Generalized Estimating Equation Models (GEE) were used to assess the factors related to the evolution of prolactin, oxytocin, and homocysteine levels. These methods analyze the evolution of a measure taking into account covariates that may be time dependent. They also take into account the correlation structure by including a working correlation matrix, increasing the efficiency of the estimators, and considering the structure of paired data throughout the follow-up. In addition, they are robust against missing data during the follow-up. Because these methods do not have goodness of fit measures, the Quasilikelihood Information Criterion was used instead. Because we intend to analyze evolution, time was introduced as a co-variable in the model regardless of whether it was a significant covariate.

The level of significance was set at α = .05 and all analyses were carried out with the IBM SPSS v.24, StatGraphics XVIII and GraphPad Prism 9 statistical packages. Exploratory analysis without a global null hypothesis are presented, thus, corrections were not made for multiple comparisons.

## RESULTS

### Demographic and Clinical Characteristics of the Cohort

The demographic and clinical characteristics of the participants in this study are presented (Table 1): 120 participants with an FEP at baseline, of whom 75 were followed-up. All participants included in the study were in the same age range. No significant differences were found in the demographic and clinical features of the patients at baseline and follow-up, except for their diagnosis, given that different proportions of nonaffective and affective psychosis were observed between the baseline and follow-up (*P *= .025: Table 1).

**Table 1.** Demographic and Clinical Characteristics of the Participants

Characteristic	Patients baseline (n = 120)	Patients follow-up (n = 75)
Age, y (mean ± SD)	23.9 ± 5.8	25.08 ± 6
Sex, n (%)		
Male	82 (68.3)	51 (68)
Female	38 (31.7)	24 (32)
Ethnic group, n (%)		
Caucasian	113 (94.2)	69 (92)
Hispanic	3 (2.5)	2 (2.7)
Others	4 (3.3)	4 (5.3)
Body mass index (mean ± SD)	24.89 ± 4.04	24.63 ± 5.1
Psychiatric history		
DUP (mean ± SD)	96.95 ± 113.79	
Diagnosis, n (%)^*^		
Affective psychosis	22 (18.3)	24 (32.4)
Nonaffective psychosis	98 (81.7)	50 (67.6)
Antipsychotic medication, n (%)		
Risperidone	43 (35.8)	15 (20.5)
Aripiprazole	12 (10.0)	15 (20.5)
Olanzapine	15 (12.5)	5 (6.8)
Quetiapine	7 (5.8)	5 (6.8)
Clozapine	8 (6.7)	5 (6.8)
Ziprasidone	2 (1.7)	2 (2.7)
Paliperidone	10 (8.3)	5 (6.8)
None	23 (19.2)	21 (28.8)
DDD of CPZ eq., (mg) (mean ± SD)	49.62 ± 127.88	39.84 ± 87.85

Abbreviations: DDD of CPZ eq., Defined daily dose of chlorpromazine equivalent; DUP, days of untreated psychosis. Comparison between FEP patients at baseline and the follow-up assessed with a *t* test (age, body mass index) a chi-squared test (gender, diagnosis, medication), the likelihood ratio (ethnic group) and a Mann Whitney U-test (DDD of CPZ eq.). ^*^P = .025.

### Plasma Prolactin, Oxytocin, and Homocysteine Levels in FEP Patients at Baseline and Follow-Up

**Figure 1.**
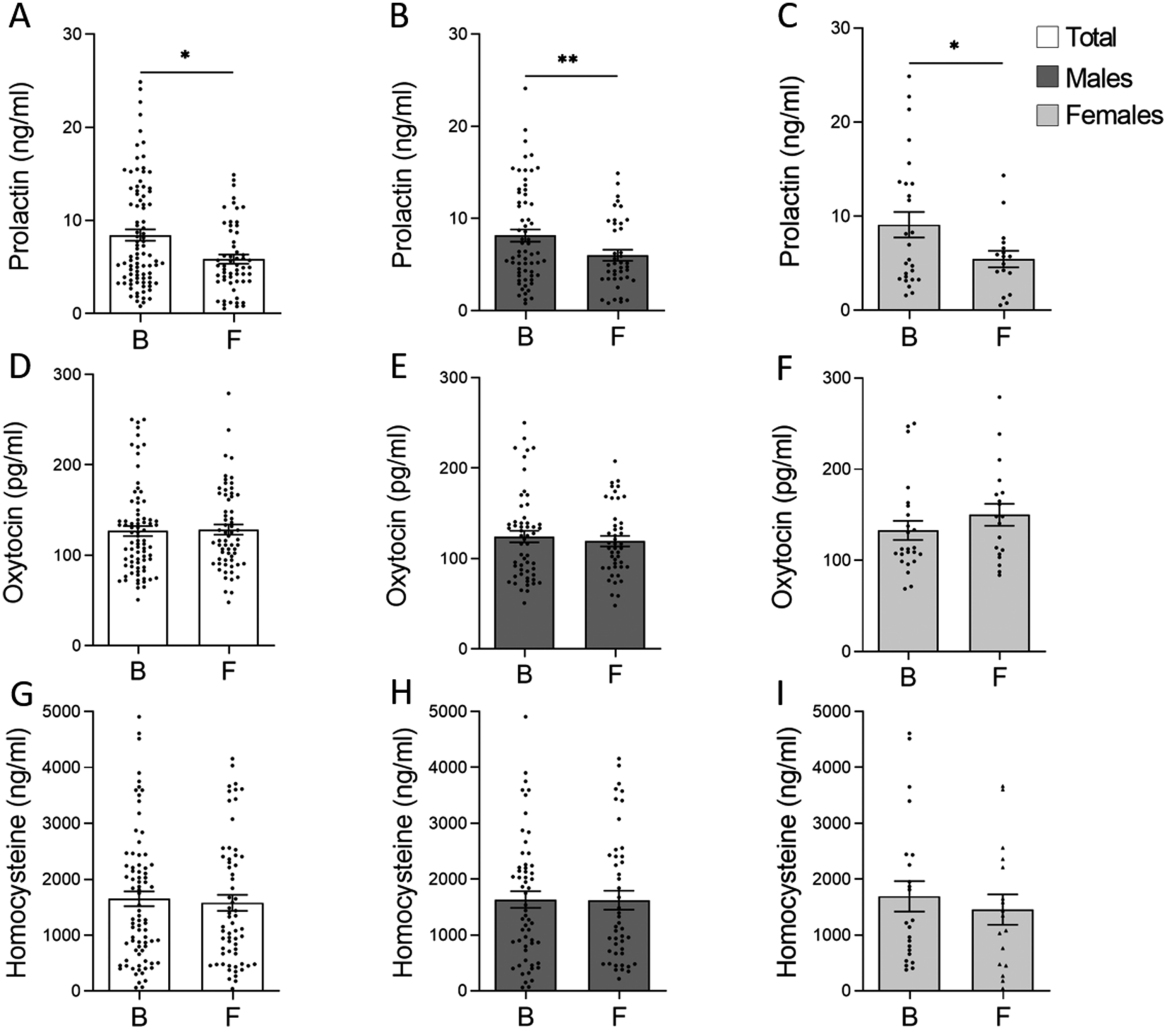
Mean differences ± SEM of the biological markers in the plasma from first-episode psychosis (FEP) patients at baseline and at follow-up. (A, D, G) Plasma prolactin (baseline n = 90; follow-up n = 58), oxytocin (baseline n = 80; follow-up n = 64) and homocysteine (baseline n = 80; follow-up n = 63) in the total group, including women and men. (B, C) Plasma prolactin in women (baseline n = 26; follow-up n = 17) and men (baseline n = 64; follow-up n = 41). (E, F) Plasma oxytocin in women (baseline n = 24; follow-up n = 19) and men (baseline n = 56; follow-up n = 45). (H, I) Plasma homocysteine in women (baseline n = 23; follow-up n = 17) and men (baseline n = 57; follow-up n = 46). A Mann-Whitney U-test or a *t* test were used to compare the groups: *P <.05; **P <.01. B, baseline; F, follow-up.

A significant decrease in prolactin was evident in patients at follow-up relative to the baseline (*P *= .017; Figure 1A). Significantly, this difference was maintained when the groups analyzed were sex disaggregated (males, *P* = .008; females, *P* = .020; Figure 1B and C). Except for 2 samples from patients at baseline, 1 male and 1 female, prolactin levels remained below 20 ng/mL in men and 25 ng/mL in women in all cases (Figure 1B and C). Hence, the higher prolactin levels in FEP patients at baseline were not considered to be hyperprolactinemia.

No change in plasma oxytocin levels were detected between the baseline and follow-up in the plasma samples (*P* = .605; Figure 1D), even when the data were sex disaggregated (males, *P* = .570; females, *P* = .289; Fig 1E and F). Similarly, there were no changes in plasma homocysteine levels between baseline and follow-up (*P* = .757; Figure 1G), as also observed when the data were sex disaggregated (males, *P* = .965; females, *P* = .549; Figure 1H and I).

**Figure 2.**
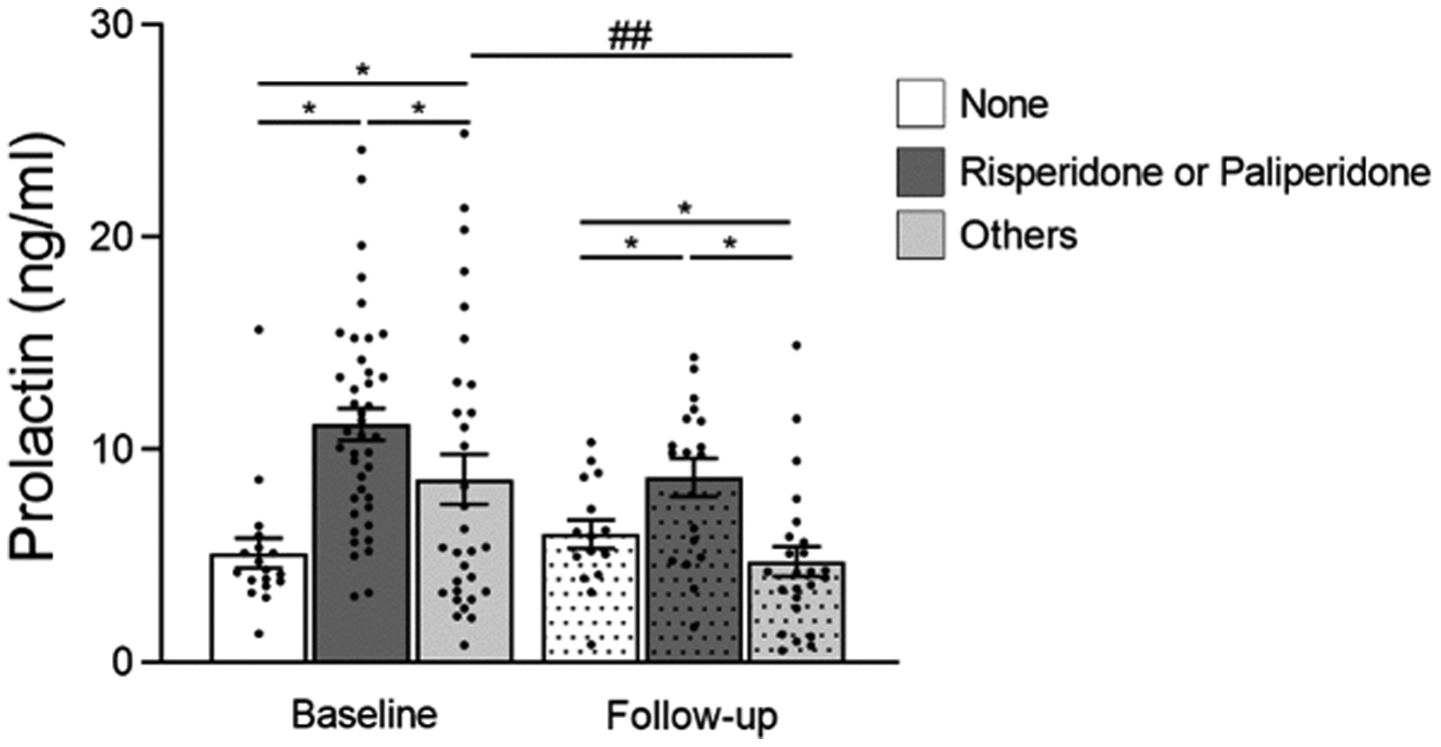
Mean differences ± SEM of plasma prolactin from first-episode psychosis (FEP) patients at baseline and at follow-up (dotted bars). Plasma prolactin from antipsychotic naïve patients (white bar, baseline n = 18; follow-up n = 15), patients treated with risperidone or paliperidone (dark grey bar, baseline n = 41; follow-up n = 18), or patients treated with other antipsychotics (grey bar, baseline n = 31; follow-up n = 24). ANOVA was used to compare between the 3 groups (baseline *P* < .001; follow-up *P* < .002), and significant post hoc differences were found between all pairs of groups at baseline or at follow-up, between none and risperidone/paliperidone, and between risperidone/paliperidone and others: *P < .05. Also, *t* tests were used to compare between the baseline and follow-up groups: ^##^*P* < .01.

The plasma samples were then divided into 3 groups depending on the antipsychotic drugs administered: (1) none, (2) risperidone or paliperidone, and (3) others. There were significant differences in the prolactin levels between the 3 groups at both baseline (*P* < .001) and follow-up (*P* = .002), with the highest levels of prolactin in the participants receiving risperidone or paliperidone (Figure 2). The lowest levels of prolactin at baseline were observed in unmedicated participants, but at follow-up, prolactin levels were even lower in patients using antipsychotics other than risperidone and paliperidone (Figure 2). Indeed, when comparing the 3 groups between baseline and follow-up, a decrease in plasma prolactin was evident at follow-up in the participants treated with antipsychotics other than risperidone and paliperidone (Group 3, *P* = .007: Figure 2). However, no differences in prolactin were observed between baseline and follow-up in unmedicated patients (*P* = .381: Figure 2) or in patients treated with risperidone or paliperidone (*P* = .059: Figure 2).

### Clinical and Cognitive Evaluation in FEP Patients

Compared with FEP patients at baseline, patients at follow-up had lower PANSS positive (*P* = .036) and PANSS general (*P = *.025) scores and an increase in the GAF score (*P = *.018; Table 2). Hence, patients at follow-up had less severe positive and general symptoms and better overall functioning. When these data were sex disaggregated, the differences between baseline and follow-up in PANSS general (*P* = .013) and GAF were evident only in men (*P* = .007; Table 2). Moreover, the PANSS total score decreased in men at follow-up relative to the baseline (*P* = .031), indicating that severity dropped between the 2 visits in men. Cognitive assessment of the patients were only performed at baseline (see Table 2).

**Table 2.** Clinical and Neuropsychological Evaluation

Evaluation	Sex	Baseline (W = 38, M = 82)	Follow-up (W = 24, M = 51)
PANSS Positive	Total	11.38 ± 6.16	9.48* ± 3.64
	Women	11.05 ± 6.46	9.58 ± 4.07
	Men	11.52 ± 6.05	9.43 ± 3.49
PANSS Negative	Total	14.44 ± 6.05	14.26 ± 6.7
	Women	14.53 ± 7.21	16.26 ± 8.43
	Men	14.4 ± 5.48	13.43 ± 5.74
PANSS General	Total	27.94 ± 10.17	24.52* ± 8
	Women	27.82 ± 12.18	25.89 ± 8.65
	Men	28 ± 9.18	23.96* ± 7.74
PANSS Total	Total	53.76 ± 19.78	48.26 ± 15.92
	Women	53.39 ± 23.45	51.74 ± 19.73
	Men	53.93 ± 17.99	46.83* ± 14.06
Overall functioning score (GAF)	Total	67.31 ± 14.35	72.4* ± 15.15
	Women	68.18 ± 15.59	70 ± 17.18
	Men	66.9 ± 12.93	73.6** ± 14.07
Verbal ability			
Vocabulary subtest (WISC-IV/WAIS-III)		94.26 ± 18.34	—
Working memory			
Digit subtests (WISC-IV/WAIS-III)		45.27 ± 8.74	—
Letter and number subtests (WISC-IV/WAIS-III)		43.53 ± 12.22	—
Processing speed			
Trail making test (Form A)		37.42 ± 20.81	—
Executive function			
Trail making test (Form B)		87.69 ± 41.42	—
Sustained attention			
Omission errors		59.86 ± 25.94	—
Commission errors		52.21 ± 11.42	—
Hit-RT		53.16 ± 10.85	—
Hit-RT-SE		58.01 ± 13.53	—
Variability		57.18 ± 13.34	—
Detectability		51.8 ± 9.68	—
Perseveration		63.79 ± 31.46	—
Hit-RT-BC		50.61 ± 11.26	—
Hit-RT-BC-SE		55.01 ± 11.08	—
Hit-RT-ISI		52.02 ± 12.87	—
Hit-RT-ISI-SE		51.33 ± 14.59	—

Abbreviations: Hit-RT, Hit reaction time; Hit-RT-BC, Hit reaction time by block; Hit-RT-BC-SE, standard error of hit reaction time by block; Hit-RT-ISI, Hit reaction time by interstimulus interval; Hit-RT-ISI-SE, standard error of hit reaction time by interstimulus interval; M, men; PANSS, Positive and Negative Syndrome Scale; W, women; WAIS, Wechsler Adult Intelligence Scale; WISC, Wechsler Intelligence Scale for Children. *Significant difference in patients between the values at baseline and follow-up as assessed with a *t* test (GAF scores) or Mann Whitney U test (PANSS scores): *P < .05. **P < .01. Bolded values: statistically significant differences. Scores (means ± SD) obtained in FEP patients.

### Correlations Between Biological Markers and Clinical Characteristics in FEP Patients at Follow-Up

Plasma prolactin levels were negatively correlated with a PANSS negative score at follow-up (rho = −0.447, *P* = .003; Table 3), suggesting that the negative symptomatology of patients with less prolactin was more severe at this stage. With sex-disaggregated data, this correlation only held for men (r = −0.492, *P* = .006; Table 3), and in men there was also a positive correlation between prolactin levels and a PANSS positive score (rho = 0.365, *P* = .047) such that men with less prolactin appeared to experience less severe positive symptoms. No significant correlation was found between plasma oxytocin levels and clinical features (Table 3), whereas plasma homocysteine levels were negatively correlated with a PANSS positive score in men at follow-up (rho = −0.35, *P* = .042; Table 3) such that men with higher levels of this biomarker at follow-up had less severe positive symptoms. No correlation with other variables was observed for homocysteine (Table 3). Moreover, no correlations were found between these molecules and PANSS general, PANSS total, the GAF scores, or antipsychotic medication at follow-up.

**Table 3.** Correlations Between Biological Markers and Clinical Features at Follow-Up

	Coefficient of correlation								
	Prolactin			Oxytocin			Homocysteine		
	Total	Women	Men	Total	Women	Men	Total	Women	Men
PANSS Positive	0.178	−0.287	0.365*	−0.167	−0.456	−0.072	−0.155	0.317	−0.35*
PANSS Negative	−0.447*	−0.433	−0.492**	−0.052	0.151	−0.199	−0.034	0.063	−0.036
PANSS General	−0.062	−0.470	0.072	−0.093	0.002	−0.206	0.027	0.214	−0.026
PANSS Total	−0.240	−0.481	−0.052	0.036	−0.010	−0.228	−0.061	0.184	−0.072
GAF	−0.009	0.049	−0.093	−0.125	−0.202	0.023	0.058	−0.072	0.021
DDD of CPZ eq.	0.058	−0.264	0.231	−0.123	−0.119	−0.176	0.098	−0.040	0.142

Abbreviations: DDD of CPZ eq., Defined daily dose of chlorpromazine equivalent; GAF, overall functioning score; PANSS, Positive and Negative Syndrome Scale. *Statistically significant correlation (Spearman or Pearson correlations: *P < .05, **P < .01).

### Generalized Estimating Equation Models (GEE)

GEE models were used to search for factors associated with the evolution of each molecule between the baseline and follow-up in FEP patients (Table 4).

**Table 4.** GEE Models

	Variable	Beta	95% CI (Beta)	*P*
GEE model 1: evolution of prolactin levels	Time (follow up vs baseline)	−34.163	(−52.385; −15.942)	**<.001**
	Hit RT ISI SE	−0.95	(−1.472; −0.428)	**<.001**
	Letter and number test	−0.962	(−1.58; −0.344)	**.002**
	DDD of CPZ eq.*	0.057	(0.017; 0.097)	**.005**
	PANSS negative*	−1.285	(−2.468; −0.101)	**.033**
GEE model 2: evolution of oxytocin levels	Time (follow up vs baseline)	0.303	(−13.559; 14.164)	.966
	Sex (female vs male)	33.391	(9.683; 57.1)	**.006**
	Hit RT ISI SE	−0.694	(−1.376; −0.012)	**.046**
GEE model 3: evolution of homocysteine levels	Time (follow-up vs baseline)	−147.131	(−437.621; 143.359)	.321
	Premorbid IQ	21.907	(2.576; 41.239)	**.026**
	Hit RT BC SE	24.844	(1.956; 47.732)	**.033**

Abbreviations: DDD of CPZ eq., defined daily dose of chlorpromazine equivalent; GEE, Generalized Estimating Equation Models; Hit RT BC SE, standard error of hit reaction time by block; Hit RT ISI SE, standard error of hit reaction time by interstimulus interval; PANSS, Positive and Negative Syndrome Scale. *These variables were time dependent.

#### GEE for Prolactin

There was a mean decrease of 34.163 points for prolactin between the baseline and follow-up. Indeed, there was a decrease of 1.285 points of prolactin for each unit increase in the PANSS negative score between the baseline and follow-up. Moreover, for each unit decrease in the daily dose of chlorpromazine equivalent, prolactin decreased 0.057 points between the baseline and follow-up. Apart from this, for each unit increase in the score of the Letter and Number test at baseline, prolactin showed a decrease of 0.962 points, suggesting that lower levels of prolactin at this stage were related to better working memory. On the other hand, for each unit of increase in the standard error of hit reaction time by interstimulus interval score at baseline, prolactin showed a decrease of 0.95 points, indicating that lower levels of prolactin were related to worse performance in attention.

#### GEE for Oxytocin

The GEE model indicated that women had higher plasma oxytocin levels than men (33.391 units more). Moreover, this molecule decreased 0.694 points for each unit increment in the standard error of hit reaction time by interstimulus interval test score at baseline, indicating that lower levels of oxytocin at this stage were related to worse performance in attention.

#### GEE for Homocysteine

An increase of 24.844 points in homocysteine was associated with an increase in each unit of the standard error of hit reaction time by block(Hit-RT-BC-SE) score at baseline, indicating a relationship between higher levels of homocysteine and worse performance in this test of attention. By contrast, better verbal ability performance was associated with higher levels of homocysteine, as there was an increase of 21.907 points for each unit increase in the Premorbid IQ score at baseline.

Although there was a difference in the percentage of diagnoses between baseline and follow-up (affective and nonaffective psychosis; Table 1), this variable was not significant in any GEE model.

## DISCUSSION

The goal of this longitudinal study of prolactin, oxytocin, and homocysteine plasma levels during an FEP, from baseline to 12 months after diagnosis, was to determine whether these molecules were associated with the clinical characteristics or neurocognitive performance of the individual. We found that plasma prolactin levels were lower at the follow-up visit than at baseline, having previously found that at baseline patients had higher prolactin levels than healthy individuals (Hidalgo-Figueroa et al., 2022). Thus, the decrease over time would appear to bring the affected individuals closer to a healthy state. Antipsychotic treatments appear to increase plasma prolactin levels, although this depends on the type of antipsychotic agent (Berwaerts et al., 2010; Peuskens et al., 2014; Wadoo et al., 2017). As indicated above, some antipsychotic agents increase prolactin whereas others do not, such that the specific antipsychotic treatment used by the patient was taken into consideration when analyzing the plasma prolactin levels. The patients treated with any antipsychotic had higher prolactin levels at baseline than untreated patients, as previously described (Hidalgo-Figueroa et al., 2022). However, prolactin levels were even higher in patients receiving paliperidone or risperidone treatment, and only patients treated with these agents had higher prolactin at follow-up relative to antipsychotic-naïve patients. By contrast, patients treated with any other antipsychotic had less prolactin, even below the levels of unmedicated patients. These data together suggest that some antipsychotics other than paliperidone or risperidone can induce a moderate increase in prolactin, especially at the beginning of the treatment (Peuskens et al., 2014). Interestingly, even unmedicated FEP patients experience an increase in prolactin compared with control participants (Garcia-Rizo et al., 2012; Riecher-Rössler et al., 2013; Petrikis et al., 2016; Delgado-Alvarado et al., 2019; Pisk et al., 2019; Studerus et al., 2021); although the underlying biological reason for this increase is unclear, the stress associated with the onset of an FEP and genetic vulnerability may influence the induction of prolactin release (Peuskens et al., 2014; Delgado-Alvarado et al., 2019; Labad, 2019). We previously, failed to observe higher prolactin levels in unmedicated patients than in healthy controls (Hidalgo-Figueroa et al., 2022), but these unmedicated patients also had less severe psychotic symptoms and better overall functioning than medicated patients (Hidalgo-Figueroa et al., 2022). Irrespective of the medication, we found a relationship between higher prolactin levels and more severe positive or general symptoms at baseline in FEP patients, specifically in males. As such, we cannot exclude the possibility that, apart from being a side effect of antipsychotics, prolactin is more specifically elevated in male patients with more severe symptoms (Hidalgo-Figueroa et al., 2022). However, it should also be noted that in patients with more severe symptoms who require higher doses of antipsychotics, higher levels of prolactin may be induced due to a greater affinity for D2 receptors (Peuskens et al., 2014). In addition, long-term exposure to antipsychotics that increase prolactin elevate the risk of breast cancer in women and of low-energy fractures (Taipale et al., 2021; Solmi et al., 2022). Thus, to mitigate high prolactin levels and any related undesired side effects, plasma prolactin levels at the beginning of an FEP should be considered before choosing an antipsychotic treatment (Peuskens et al., 2014).

Regarding the implication of the oscillations in prolactin and the clinical status of the patients, a GEE model indicated that patients in whom prolactin decreases from baseline to the follow-up had worse negative symptoms, together with a lower antipsychotic dose. In addition, there was a correlation between low levels of prolactin at follow-up and more severe negative symptoms in male patients. With respect to positive symptoms, there was a positive correlation between prolactin levels and the PANSS positive score at baseline (Hidalgo-Figueroa et al., 2022) and at the follow-up (data herein), specifically in men, in concordance with data presented elsewhere (Delgado-Alvarado et al., 2019; Pisk et al., 2019). By contrast, higher prolactin levels in FEP female patients were negatively correlated with the severity of positive symptoms, suggesting prolactin could serve as a protective factor in females (Delgado-Alvarado et al., 2019). Together, prolactin levels may be associated with positive and negative symptomatology in a sex-dependent manner.

Through the GEE model, we also found that prolactin levels had a distinct influence on the cognitive domains assessed at baseline, whereby lower prolactin was related to better working memory and worse sustained attention. However, we did not observe an influence of sex on the relationship between prolactin and cognition when controlling for the rest of the covariates in this GEE model. Increased prolactin has been related to worse cognitive performance elsewhere, more specifically in processing speed and exclusively in FEP male patients (Montalvo et al., 2014, 2018), suggesting a sex-dependent effect.

In terms of oxytocin, we previously showed that FEP patients had lower plasma oxytocin levels at baseline than healthy controls (Hidalgo-Figueroa et al., 2022), and here we found there were no differences in oxytocin levels between patients at baseline and follow-up. When plasma oxytocin levels were previously measured in patients with schizophrenia, conflicting results were obtained, that is, lower or higher levels of oxytocin or even no change relative to healthy participants (Beckmann et al., 1985; Glovinsky et al., 1994; Goldman et al., 2008; Strauss et al., 2019). This variability may reflect the characteristics of the participants studied, such as sex, symptom severity, medication, or the stage of the disorder (Rubin et al., 2010, 2011, 2013; Sasayama et al., 2012; Kirsch, 2015). Regarding sex, we found female patients had higher levels of oxytocin than males, although this difference did not affect the association between oxytocin level and clinical characteristics because no correlations were detected between oxytocin and the PANSS or GAF scores at any of the stages analyzed. Indeed, no relationship between plasma oxytocin levels and clinical symptoms was seen in an earlier study on FEP patients (Rubin et al., 2013). By contrast, low levels of oxytocin were related to the severity of symptoms in patients with schizophrenia, especially to negative and cognitive symptoms (Shilling and Feifel, 2016), leading to the proposal of exogenously administering oxytocin to manage these symptoms (Kirsch, 2015; Shilling and Feifel, 2016). Medication may also influence oxytocin levels because second-generation antipsychotics have been associated with lower levels of oxytocin in patients with schizophrenia (Sasayama et al., 2012). However, we did not observe any correlation between the daily equivalent dose of chlorpromazine and oxytocin in FEP patients at either baseline (Hidalgo-Figueroa et al., 2022) or follow-up.

Regarding cognitive performance at baseline, the GEE model for oxytocin showed that lower levels of oxytocin at this stage were related to worse performance in sustained attention. We previously reported a correlation in the same sense (as evaluated with the perseveration test) but only in women because low oxytocin levels in men were correlated to better sustained attention performance (as assessed by omission errors: (Hidalgo-Figueroa et al., 2022). However, other studies reported no relationship between oxytocin and cognitive performance in FEP patients (Rubin et al., 2013), or, conversely, there was a connection between low levels of oxytocin and impaired cognitive performance in patients with schizophrenia, mainly in males (Strauss et al., 2019). When first evaluated, oxytocin treatment of patients at early psychosis did not produce improvements, although it did appear to be related to an improvement in negative symptoms (Cacciotti-Saija et al., 2015). Together, the differences in the results between schizophrenia and FEP may suggest that the stage of psychosis influences the relationship between oxytocin and medication or clinical/cognitive symptomatology. As such, perhaps early modulation of oxytocin would improve the outcome of patients at high risk of evolving to schizophrenia. Although sex did not influence our GEE model for oxytocin, it would be interesting to further analyze the effect of sex in the relationship between endogenous oxytocin and clinical or cognitive performance, which may help better design clinical trials based on the use of oxytocin as a supplementary treatment in schizophrenia (Bradley and Woolley, 2017).

Plasma homocysteine levels did not change in FEP patients between baseline and follow-up, yet we found several associations between homocysteine levels and symptomatology in these patients. At baseline, a GEE model indicated that higher homocysteine levels were associated with worse performance in sustained attention and better verbal abilities. A recent clinical trial showed that supplementation with vitamin B decreases homocysteine levels, improving attention/vigilance performance, particularly in patients with initially elevated homocysteine levels, females, and individuals with affective psychosis (Allott et al., 2019). Indeed, previous studies reported that high plasma homocysteine levels represent a potential risk factor to suffer a FEP or schizophrenia (Muntjewerff et al., 2006; García-Bueno et al., 2014) and that elevation of this molecule is associated with the severity of negative symptoms (Petronijević et al., 2008; Fan et al., 2017). Interestingly, that association was inverted after 3 months of treatment with risperidone (Fan et al., 2017). Here, higher homocysteine levels during the follow-up were correlated with less severe positive symptoms in male patients alone. It was recently reported that homocysteine produced in erythrocytes may contribute to intracellular antioxidant capacity under oxidative stress, which may be a protective factor against reactive oxygen species (Ye et al., 2023). Further analyses will be needed to evaluate whether decreasing homocysteine could be beneficial depending on patient’s sex and clinical/cognitive characteristics (Allott et al., 2019).

There are some limitations to the present study, not least that 45 patients were not followed-up throughout the study and, thus, the sample size at follow-up was reduced with respect to the baseline, which may affect the comparison between the groups. In addition, the number of men included in the study was approximately double that of women, which could influence the results obtained. Indeed, some sex-disaggregated analyses could not be carried out due to the small number of women, such as the analysis of the effect of different antipsychotics on prolactin levels. Finally, to better understand the relationship between prolactin, oxytocin, and homocysteine levels and clinical/cognitive variables, longer follow-up periods and cognitive evaluations at each visit would be necessary. Nevertheless, one of the strengths of the study is the type of patient, FEP patients, which favors the search for factors associated to this disorder and avoids confounding factors, like long diseases duration or long periods of drug therapy. A further strength is the in-depth cognitive evaluation to assess different domains. In addition, it is useful that sex-disaggregated analyses were performed to understand the influence of sex on our measurements.

Together, our data suggest that plasma prolactin, oxytocin, and homocysteine levels may be related to cognitive performance of FEP patients at baseline, particularly in terms of working memory, verbal ability, and sustained attention domains. Plasma prolactin and homocysteine levels could be associated with psychotic symptomatology in FEP patients, specifically in men. Interestingly, clinical improvement during the follow-up, together with the type and dose of antipsychotic used are associated with prolactin levels, suggesting it may be useful to consider prolactin levels when choosing the medication for these individuals, which may differ between sexes. In summary, studying prolactin, oxytocin, and homocysteine from the onset of an FEP may help improve the pharmacological treatment and outcome of these patients, bearing in mind the sex of the individual in all these studies.

## Data Availability

The data underlying this article will be shared on reasonable request to the corresponding author.

## Interest Statement

In addition to having received competitive funding, several authors have received honoraria: Juan C. Leza, as consultant or speaker from Lundbeck, Otsuka, Alicia Koplowitz Foundation, Sanofi, ChemoLab-Liconsa, Quantum, Janssen-Cilag, Ferrer, Bayer, and Sanofi. M. Bernardo consultant or speakers/advisory board of ABBiotics, Adamed, Angelini, Casen-Recordati, Janssen-Cilag, Menarini, Rovi, and Takeda. A. Gonzalez-Pinto as consultant or offered lectures from Janssen-Cilag, Lundbeck, Otsuka, Pfizer, Takeda, and Angelini. M. P. García-Portilla as consultant or speaker from Alter, Angelini, Cassen-Recordati, Janssen-Cilag, Idorsia, Lundbeck, Otsuka, and SAGE Therapeutics. M. Bioque as consultant or talks for Adamed, Angelini, Casen-Recordati, Exeltis, Ferrer, Janssen, Lundbeck, Neuraxpharm, Otsuka, Pfizer, and Sanofi. R. Rodríguez-Jiménez as consultant or speaker from Janssen-Cilag, Lundbeck, Otsuka, Pfizer, Ferrer, Juste, Takeda, Exeltis, Casen-Recordati, Angelini, and Rovi. M. Parellada has received grant support from Fundación Alicia Koplowitz, Fundación Familia Alonso, and Fundación Mutua Madrileña and has received honoraria as speaker or consultant from Exeltis, Servier, Roche, and Janssen.
